# Impact of data compression and security devices on telesurgery systems

**DOI:** 10.1007/s00595-025-03142-7

**Published:** 2025-10-07

**Authors:** Hajime Morohashi, Kenichi Hakamada, Yusuke Wakasa, Kazuki Yokoyama, Yuma Ebihara, Satoshi Hirano, Eiji Oki, Norihiko Ikeda, Akinobu Taketomi, Masaki Mori

**Affiliations:** 1 Committee for Promotion of Remote Surgery Implementation, Japan Surgical Society, Tokyo, Japan; 2 Department of Gastroenterological Surgery, Hirosaki University Graduate School of Medicine, 5 Zaifu-cho, Hirosaki-shi, Aomori 036-8562 Japan; 3 Department of Gastroenterological Surgery II, Faculty of Medicine, Graduate School of Medicine, Hokkaido University, Sapporo, Japan; 4 Center for Integration of Advanced Medicine, Life Science and Innovative Technology (CAMIT), Kyushu University Hospital, Fukuoka, Japan; 5 Department of Gastroenterological Surgery I, Hokkaido University Graduate School of Medicine, Sapporo, Japan; 6 Department of Surgery, Tokyo Medical University, Tokyo, Japan; 7 Tokai University School of Medicine, Isehara, Japan

**Keywords:** Telesurgery, Remote surgery, Robotic surgery, Communication delay, IPsec

## Abstract

**Purpose:**

To evaluate the feasibility of secure telesurgery by assessing the impact of image compression and cybersecurity devices on surgical performance and data transmission.

**Methods:**

Telesurgical procedures using the *hinotori™* surgical robot were performed remotely between Hirosaki and Goshogawara, which are 30 km apart, over a secure line provided by NTT East. Image compression was tested at 120, 80, 60, 40, and 20 Mbps. A surgical specialist operated on artificial organ models. Simulated cyberattacks were introduced to assess the performance of security devices.

**Results:**

Even at 20 Mbps, there was no significant loss in operability or image quality. Security devices detected simulated attacks and permitted essential robot communications. No visual distortion or operational issues occurred, and only a small delay of ≤ 2 min was introduced. The transmission control protocol (TCP) error rates remained low, with or without security devices (0.00–0.04%).

**Conclusion:**

Security implementation enables safe telesurgery by detecting cyber threats without impairing surgical performance. This finding supports the practical development of economically and technologically viable telesurgical systems.

## Introduction

With advances in robot-assisted surgery, telesurgery has emerged as a promising technology that enables the delivery of advanced medical care across geographical boundaries. The robot-assisted telesurgery performed between France and the United States in the early 2000 s was a milestone in this field, gaining worldwide attention as the first international telesurgical procedure. This pioneering achievement became a benchmark for subsequent technological developments in telesurgery [1]. In Japan, research into the practical implementation of telesurgery is progressing rapidly, driven by a shortage of surgeons and regional disparities in access to medical care.

We have conducted demonstration experiments of remote robotic surgery using domestic commercial communication lines and domestically manufactured robots in various regions of Japan, including Hokkaido, Aomori Prefecture, Kobe City, and Kyushu. These trials allowed us to report the successful establishment of a system enabling telesurgery within Japan [2–5]. Furthermore, we conducted extensive experiments on essential aspects of telesurgery, including robotic technology, required communication bandwidth, acceptable latency thresholds, data processing for communication, and communication security. These studies yielded a range of important research findings that have contributed to the practical realization of telesurgery [6–9]. Based on these results, it is now considered viable to perform remote surgery using domestic communication lines, although challenges remain in terms of economic feasibility and safety. In general, telesurgery requires high-bandwidth communication, and the use of closed, dedicated lines is costly, making the current system economically unsuitable for widespread social implementation. One potential solution is to reduce the volume of transmitted data, thereby enabling the use of high-quality, lower-cost communication lines.

The safety of telesurgery depends not only on the sophistication of robotic and endoscopic technologies, but also on the quality and reliability of the communication infrastructure [10]. If low-latency, high-bandwidth communication lines are not secured adequately, if redundancy for emergency response is lacking, or if countermeasures against unauthorized access are insufficient, telesurgery systems may face situations that pose direct threats to patient safety. With respect to cybersecurity, there is a significant risk that medical data and robotic control signals could be intercepted or tampered with by external malicious interference. This makes security a critical issue that must be addressed for the safe and reliable implementation of telesurgery in society [1, 4].

Reports suggest that safety can be ensured by implementing a redundant system using a secondary communication line to mitigate the risks associated with communication interruptions [7, 9]. Furthermore, Oki et al. demonstrated that incorporating IPsec encryption into an IP-VPN does not result in a substantial degradation of communication quality, highlighting its feasibility as a security measure for telesurgical systems [11]. However, there have been no reports investigating the use of security devices to counteract interference or attacks on communications during telesurgery. The purpose of this study is to evaluate the effects of data compression on transmission communication in remote robotic systems, and to assess the impact of security devices in preventing communication attacks during surgery. Ultimately, this study aims to develop a telesurgery system that offers both high economic efficiency and robust safety.

## Materials and methods

### Telesurgical system

**Fig. 1:**
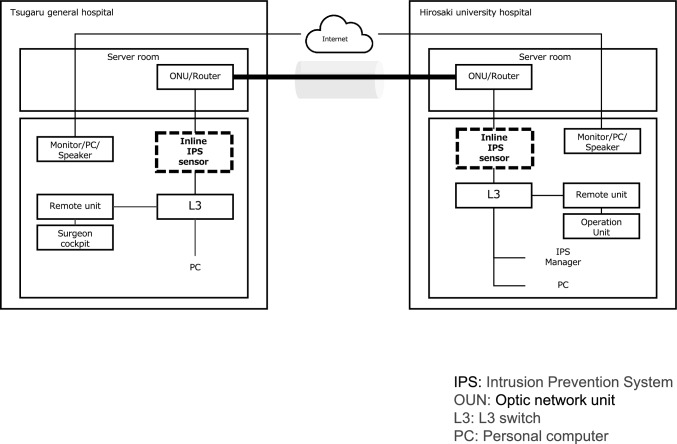
Network system (Inline type). *IPS* Intrusion Prevention System, *OUN* Optic network unit, *L3* L3 switch, *PC* Personal computer

**Fig. 2:**
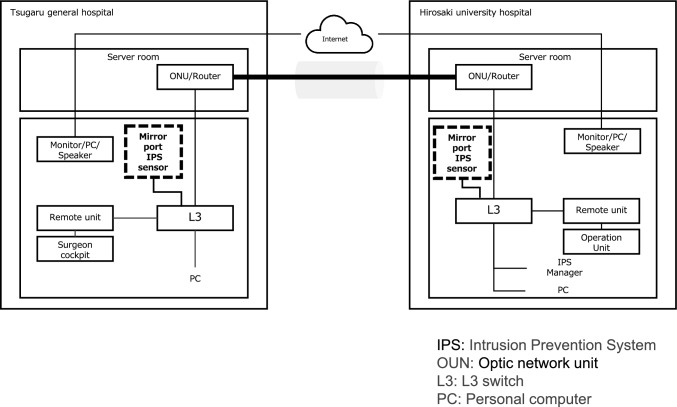
Network system (Mirror port type). *IPS* Intrusion Prevention System, *OUN* Optic network unit, *L3* L3 switch, *PC* Personal computer

The surgical robot used in this study was the *hinotori*, developed by Medicaroid. The Operation Unit was installed at Hirosaki University Hospital in Hirosaki City, and the Surgeons’ Cockpit was located approximately 30 km away at Tsugaru General Hospital in Goshogawara City. The two sites were connected via communication lines provided by NTT East Japan. Three types of communication lines were employed: Best Effort 1 Gbps (Managed SD-WAN), Guaranteed 100 Mbps line (Interconnected WAN), and IOWN 1 Gbps (All-Photonics Connect powered by IOWN). For data transmission, image data were compressed using variable encoder settings to achieve bitrates of 120 Mbps, 80 Mbps, 60 Mbps, 40 Mbps, and 20 Mbps. To evaluate security-related performance, simulated security threats were introduced via inline-type devices (Fig. 1) and mirror-port-type devices (Fig. 2), and then communication delay and image quality were assessed. Telesurgery was performed on an artificial organ model by a surgical specialist, and assessments were conducted on system operability and image degradation. This study was approved by the Committee of Medical Ethics of Hirosaki University Hospital (Approval Number: 2024–854).

### Telesurgical tasks

Five remote surgeons participated in the telesurgery sessions. Telesurgical support and telementoring were conducted in four separate surgeon pairs. Remote robotic surgery was performed on an artificial organ model developed at Hirosaki University specifically for simulating pancreatic-intestinal anastomosis [12]. One surgeon (Surgeon #1) performed the procedure using a Best-effort 1 Gbps line, three surgeons (Surgeons #2, #4, and #5) used a Guaranteed 100 Mbps line, and one surgeon (Surgeon #3) used the IOWN 1 Gbps line. Each surgeon conducted simulated telesurgery for approximately 60 min using their assigned communication line. During the procedure, the compression rate of the transmitted image data was varied within the same line to evaluate the impact of different transmission settings on surgical performance.

Table 1 summarizes the communication lines that were used and the items that were evaluated in each setting. Subsequently, each of the four remote surgeons (Surgeons #1–#4) provided remote surgical support and guidance to a local surgeon. Educational tools were utilized during these sessions, including annotation-based surgical guidance and a handover function that enabled surgeons to swap for continuous support. Image quality was assessed using the Image Quality Score (IQS) [13], as shown in Table 2a. Robot operability was evaluated using the Modified System Usability Scale (mSUS), based on the original scale developed by Brooke [14] (Table 2b), as well as the Robot Usability Score (RUS) [15] (Table 2c).

**Table 1:** Availability and verification

	Line	Security product	Compressed image data				
			120 Mbps	80 Mbps	60 Mbps	40 Mbps	20 Mbps
# 1	Best effort	In line	●	●	●	●	●
	1G						
# 2	Guaranteed	In line	–	–	●	●	●
	100 Mbps						
# 3	IOWN	In line	◯	◯	●	●	●
	1 Gbps						
# 4	Guaranteed	–	–	–	●	●	●
	100 Mbps						
# 5	Guaranteed	Mirror port	–	–	●	●	●
	100 Mbps						

◯: Usable and verified (−)

●: Usable and verified (+)

**–**: Cannot be used

**Table 2:** Evaluation criteria

a					
Image Quality Score (IQS)					
Question	Poor			Good	
How clear were the images from the robotic surgery?	1	2	3	4	5
Were the images from robotic surgery 3D?	1	2	3	4	5
Were the images from the robotic surgery clear?	1	2	3	4	5
Were the images of the robotic surgery continuous?	1	2	3	4	5
Were you able to perform surgery using images from robotic surgery?	1	2	3	4	5

b					
Modified System usability scale (mSUS)					
Question	Poor			Good	
I would like to use this system more often	1	2	3	4	5
I found the system is simple and clear to use	1	2	3	4	5
I found the system easy to use	1	2	3	4	5
I don’t think that I would need the support of a technician to use this system I no time	1	2	3	4	5
I think most people will be able to use this system in no time	1	2	3	4	5
I was able to use this system with confidence	1	2	3	4	5
This system is intuitive and easy to use	1	2	3	4	5
I found the system is very useful	1	2	3	4	5
I feel that this system helped me in performing my tasks	1	2	3	4	5

c					
Robot Usability Score (Max 40)					
Question	Poor			Good	
I was physically comfortable	1	2	3	4	5
The instrument had good hand control	1	2	3	4	5
The instrument had good foot consoles	1	2	3	4	5
The instrument had good 3D vision	1	2	3	4	5
I was not feeling stressed or annoyed by the instrument	1	2	3	4	5
This robot worked smoothly	1	2	3	4	5
This robot worked exactly as I wanted it to do	1	2	3	4	5
I wound be able to perform real operation using this robot	1	2	3	4	5

*IQS* Image quality score, *mSUS* Modified version of the system usability scale created by Brooke (mSUS) Robot, *RUS* Usability score

### Security devices

Security products utilized the Intrusion Prevention System (IPS) provided by TXOne and were deployed in an inline configuration across all communication lines. Subsequently, IPsec was added to the mirror port configuration on the Guaranteed 100 Mbps line. A network port scan was conducted from a computer located at Tsugaru General Hospital to a computer located at Hirosaki University Hospital (Fig. 1).

In the mirror port configuration, the uplink ports from each facility’s Layer 3 (L3) switch to the router were mirrored, allowing for the monitoring of inbound and outbound communications from the remote units at each site (Fig. 2). Simulated cyberattacks were introduced during remote robotic surgical tasks to evaluate the types of communications detected by the security devices. The evaluation parameters included threat detection capability, communication delays, and error packet rates, along with subjective assessments by the operating surgeons.

## Results

- (1) Verification of image compression and required bandwidth

For telesurgery using the *hinotori™* surgical robot, we developed a communication system incorporating an Intrusion Prevention System (IPS) and image data compression. The Best-effort communication line (SD-WAN) was found to be practical when image compression reduced the bitrate from 120 to 20 Mbps. The Guaranteed 100 Mbps line was excluded from the initial verification at 120 Mbps because of its insufficient bandwidth capacity. Even at 80 Mbps, the robotic system did not operate stably. This instability was attributed to limited communication bandwidth, as the total required bandwidth, including image transmission of the surgical field, robot control signals, voice communication, and IPS data, was estimated at 15–20 Mbps.

In preliminary tests, stable operation was confirmed at approximately 75 Mbps of total bandwidth usage. Based on these findings, it was concluded that the Guaranteed 100 Mbps line should have a buffer of at least 25% over the actual expected bandwidth to ensure stable performance. Although the IOWN 1 Gbps line offers extremely high bandwidth and supports various compression rates, only 60 Mbps, 40 Mbps, and 20 Mbps were evaluated in this study, all of which demonstrated stable operation without any issues.

- (2) Operability and image degradation as assessed by remote surgeons

In telesurgery performed using the verified communication system, none of the participating surgeons reported any difference in robot operability with image compression within the same communication line and confirmed the feasibility of telesurgery (Table 3). Moreover, the surgeons who operated with security devices in place (#1, #2, #3, and #5) noted no issues related to image degradation that would impair telesurgical performance.

**Table 3:** Image compression and impact on surgery

Surgeon	Line	Security product	*Excerpted questions	Compressed image data				
				120 Mbps	80 Mbps	60 Mbps	40 Mbps	20 Mbps
# 1	Best effort 1G	In line	I was physically comfortable	5	3	5	5	3
			I wound be able to perform real operation using this robot	4	4	4	4	4
# 2	Guaranteed 100 Mbps	In line	I was physically comfortable	-	-	5	3	4
			I wound be able to perform real operation using this robot					
				-		5	3	4
# 3	IOWN 1 Gbps	In line	I was physically comfortable	-	-	5	4	4
			I wound be able to perform real operation using this robot					
				-	-	5	4	4
# 4	Guaranteed 100 Mbps	-	I was physically comfortable	-	-	5	4	4
			I wound be able to perform real operation using this robot					
				-	-	5	4	4
# 5	Guaranteed 100 Mbps	Mirror port	I was physically comfortable	-	-	5	4	4
			I wound be able to perform real operation using this robot	-	-	5	4	4

^*^Excerpted Questions: Excerpts from the Robot Usability Score

**Fig. 3:**
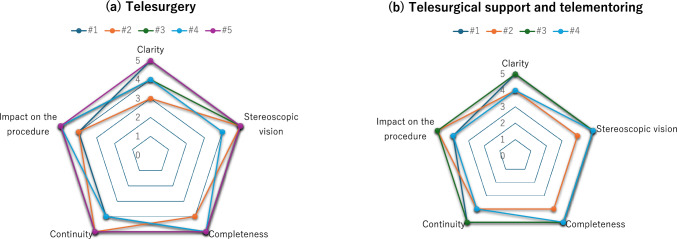
Image quality score. **a** Telesurgery. **b** Telesurgical support and telementoring

Table 4 presents the evaluation results for the Image Quality Score (IQS), the Modified System Usability Scale (mSUS), and the Robot Usability Score (RUS). No differences in operability or image quality were observed based on the type of communication line or the presence of security devices. A detailed analysis of the IQS scores revealed that all evaluated categories, including image clarity, stereoscopic perception, completeness, continuity, and impact on surgical technique, were rated 3 or higher, indicating acceptable performance with no critical issues (Fig. 3a). Results from telesurgical support and telementoring sessions, which utilized annotation tools and the surgeon handover (swapping) function, received scores of 4 or higher in all cases, further confirming satisfactory usability (Fig. 3b).

- (3) Communication attacks and security device detection

**Table 4:** Result of evaluation criteria

Surgeon	Line	Security product	IQS (Max 25)	mSUS (Max 45)	**RUS (Max 40)**
# 1	Best effort 1G	In line	23	26	29
# 2	Guaranteed 100 Mbps	In line	17	23	28
# 3	IOWN 1Gbps	In line	24	35	32
# 4	Guaranteed 100 Mbps	-	23	31	33
# 5	Guaranteed 100 Mbps	Mirror port	25	39	40

*IQS* Image quality score, *mSUS* Modified version of the system usability scale created by Brooke, *RUS* Robot usability score

To simulate a scenario in which a computer becomes infected with malware, a port scanning attack was conducted during telesurgery. The simulated attack was detected by the security device as abnormal communication; however, the communication data essential for robotic operation during the telesurgical procedure was not flagged as abnormal. It is important to note that the security device failed to detect certain communication packets related to the surgical robot. This limitation is presumed to be due to either the technical specifications of the security product or the unique communication protocols utilized by the surgical assistance robot.

- (4) Communication delays and error packets

**Fig. 4:**
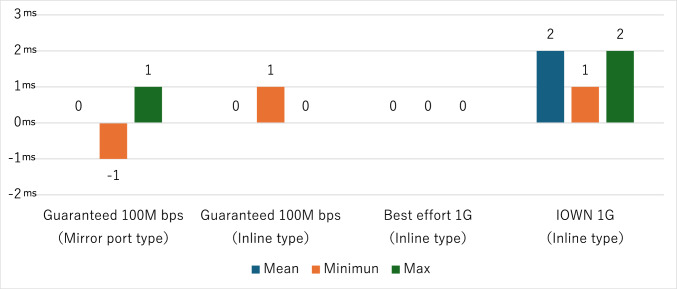
Communication delay when using security devices

Communication delay was calculated by measuring the difference in latency before and after the security device for each communication line and was defined as the delay introduced by the security device. In the inline-type configuration, the measured delay was 1 ms for the Guaranteed 100 Mbps line, 0 ms for the Best Effort line, and 2 ms for the IOWN 1 Gbps line (Fig. 4). In the mirror port configuration, the delay was 0 ms across all lines.

**Fig. 5:**
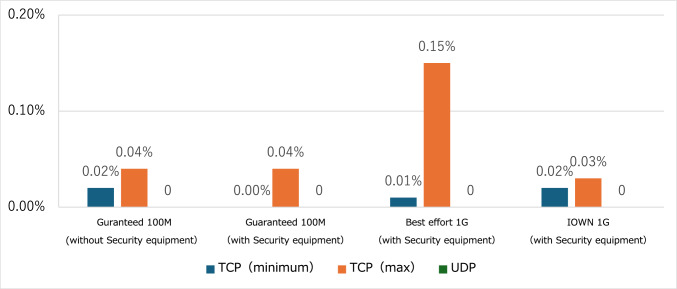
Packet loss. *TCP* Transmission Control Protocol, *UDP* User Datagram Protocol

The error packet rates were as follows: 0.04% for the Guaranteed 100 Mbps line, 0.15% for the Best Effort line, and 0.03% for the IOWN 1 Gbps line. On the Guaranteed 100 Mbps line, Transmission Control Protocol (TCP) communication error packet rates ranged from 0.02 to 0.04% without the intervention of a security device. When a security device was introduced, the error rate ranged from 0.00 to 0.04%, indicating no significant difference (Fig. 5). The maximum error packet rate for the Best Effort line was 0.15%, whereas it was 0.03% for the IOWN 1 Gbps line.

## Discussion

In this study, compressing image data to 20 Mbps had minimal impact on telesurgical operability and image quality. The security devices detected simulated communication attacks as abnormal traffic while correctly identifying the communication data essential for robotic operation as normal. The installation of these security devices did not cause any image distortion or interference with robot functionality. The additional communication delay introduced by the security equipment was at most 2 ms, indicating that its presence had no adverse effect on telesurgical performance. However, it was considered important to evaluate the impact of security equipment on overall bandwidth requirements.

Ebihara Y et al. reported that teleoperation systems using the *hinotori™* robot require communication bandwidths of at least 150 Mbps for optimal performance. When the available bandwidth is insufficient, image compression has been shown to be an effective method to avoid degradation in image quality [5]. In the pursuit of economically practical communication lines, one viable approach is to reduce transmission traffic by avoiding the need to transmit full, uncompressed data. Since image data constitutes the majority of transmitted information in telesurgery, Takahashi investigated the extent to which image compression can be applied without compromising surgical performance. Their study found that even with compression down to 10 Mbps, there was no negative impact on surgery in a telesurgical environment [14]. In this study, the surgeons’ cockpit and operation unit were physically connected via a communication line between two geographically remote cities. It was confirmed that the transmission image data could be effectively compressed, and that telesurgical performance was not adversely affected even at a bitrate of 20 Mbps. This is largely attributable to the use of optimized compression techniques, including selectively reducing image data in the peripheral regions of the display rather than applying uniform compression, as well as decreasing the amount of color information within the image.

Telesurgery has advanced remarkably over the past two decades, driven by major developments in surgical assistive robotics and communication technologies [15]. However, alongside its clinical potential, serious concerns have emerged regarding cybersecurity and the reliability of communication networks [16]. Telesurgery systems are vulnerable to a range of cyber threats, including eavesdropping, sabotage, data tampering, and unauthorized access. Ensuring the confidentiality, integrity, and availability of data is essential, as patient information and surgical commands are transmitted and received in real time [13]. Because cyberattacks and communication failures during telesurgery can compromise patient safety, the establishment of robust security measures is essential. In this study, the Intrusion Prevention System (IPS) used for verification consisted of a sensor and a manager unit. The sensor was installed at Tsugaru General Hospital, while the manager was located at Hirosaki University Hospital.

The verification involved two configurations: an inline type, which handles a high volume of communication traffic, and a mirror port type, which monitors a lighter load. Port scanning tests were conducted using the mirror port configuration. When inline IPsec was deployed in areas where unauthorized communications could potentially be monitored nearby, simulated cyberattacks were introduced to observe the effects. These tests revealed no delays or communication interruptions that would interfere with telesurgical operations.

Verification of system performance with IPsec encryption between dual surgeon cockpits demonstrated that the change in communication bandwidth was approximately 5 Mbps, with no increase in latency. No difference in error packet rates was observed with or without security devices. Although the inability to detect robot-specific communication packets in this study represents a major concern, addressing this issue with general-purpose security devices is made challenging by trade secret constraints regarding robot-specific communication protocols. Therefore, the development of security solutions tailored to each robotic system will likely become necessary. Communication protocols used in telesurgery must meet stringent requirements, including low latency, high reliability, and strong encryption. To safeguard telesurgery systems from unauthorized access, it is essential to implement real-time cybersecurity monitoring, anomaly detection, and the rapid identification of attack indicators.

The Japanese Telesurgery Guidelines recommend the use of closed communication networks: physically or logically isolated from the Internet, and the application of encryption between communication terminals when performing telesurgical procedures. These measures are intended to enhance both the security and reliability of telesurgical systems [17]. However, as the adoption of telesurgery expands, it is anticipated that a variety of connection types, including open networks both within and between facilities, will be utilized. This shift introduces new challenges for implementing institutions, particularly regarding organizational frameworks and the need for technical safeguards such as external connection management.

To ensure secure implementation, it is essential to categorize necessary security measures based on several criteria: inter-facility connectivity, maintenance and management connectivity, the physical and digital infrastructure of the facility, and the use of cloud-based services. While closed communication networks are generally considered more resilient against external cyber threats, it remains crucial to establish comprehensive security protocols, especially in light of the potential future use of open or hybrid communication networks that may not rely on closed lines.

In today’s environment of escalating cyberattacks, it is essential to prepare for both known and unknown threats. There is a pressing need to integrate cutting-edge cybersecurity technologies into the medical field, particularly in areas such as telesurgery. From the patient’s perspective, the absence of a surgeon at their bedside, combined with fears of data breaches or hacking, may lead to skepticism or mistrust toward telemedicine technologies overall. To address these concerns, a multifaceted approach is required. This should include the transparent disclosure of expert-led security assessments, the widespread dissemination of official guidelines, and public awareness initiatives aimed at building trust and understanding of the safety and reliability of remote medical technologies. Ensuring security in telesurgery is not solely a technical challenge, it is a multidisciplinary issue that spans engineering, medicine, law, and ethics [18]. Moreover, non-technical factors such as infrastructure investment by telecommunication carriers, regional disparities in network availability, and disaster resiliency pose barriers to achieving secure telecommunication for telesurgery. Therefore, national-level support for the development and strengthening of telecommunication infrastructure is essential.

This study has several limitations. First, statistical validation was not feasible because of the small number of participants assigned to each communication line type. Moreover, some variables could not be fully assessed because of measurement constraints. It was anticipated that network performance might be affected by internal communication pathways within the hospital, such as routers and intermediary devices; however, a detailed investigation of these factors was not possible. The VLANs of the surgical support robot and the malware-infected PC were configured on separate network segments, which may have prevented accurate detection of certain communication abnormalities. Furthermore, since an actual operating room was used for the study, there were limitations regarding the availability of the operating room and study location. Finally, a detailed economic analysis of the communication infrastructure was not conducted due to scope and time constraints. Communication costs are variable depending on social circumstances and corporate philosophy, making it difficult to specify specific amounts in this paper.

In conclusion, this study demonstrated that IPS-based security enhancement is an effective approach for the social implementation of telesurgery, as it enables the detection of anomalous communications without compromising the safety or performance of telesurgical operations. Additionally, the feasibility of selecting cost-effective communication lines through image compression was confirmed. Looking ahead, it will be essential to establish a sustainable operational model for remote robotic surgery, which integrates legal frameworks, medical ethics, and ongoing technological innovation. Achieving this goal will require strategic and coordinated efforts through collaboration among industry, government, academia, and the medical community.
